# A Registry-Based Perspective of Interventional Clinical Trials for Duchenne Muscular Dystrophy

**DOI:** 10.7759/cureus.115865

**Published:** 2026-09-06

**Authors:** Jeet H Chaudhary, Jiya O Chacko, Viswabhaskar Susarla, Sai Subramanya Kashyap Peraboina, Shrabya Sen

**Affiliations:** 1 Neurology, Smt. N.H.L. Municipal Medical College, Ahmedabad, IND; 2 Neurology, Sri Uthradom Thirunnal Institute of Medical Sciences, Thiruvananthapuram, IND; 3 Neurology, Kids Neuro Care LLC, Orlando, USA; 4 Neurology, Bukhara State Medical Institute, Bukhara, UZB; 5 Neurology, Rangpur Community Medical College, Kalispell, USA

**Keywords:** clinical trials, duchenne muscular dystrophy, intervention type, study design, trial phase

## Abstract

Background and objective

Duchenne muscular dystrophy (DMD) is a severe, progressive, X-linked genetic disorder caused by pathogenic variants in the dystrophin gene, leading to progressive muscle weakness, loss of ambulation, and premature mortality. Clinical trials are essential for advancing evidence-based management of DMD; however, the overall patterns of study design, intervention strategies, funding, and results reporting remain incompletely characterized. This study aimed to characterize interventional DMD clinical trials registered in ClinicalTrials.gov as of May 25, 2026, and to assess patterns in study design, intervention type, trial phase, funding, results reporting, and trial start era.

Methods

A retrospective registry-based descriptive study was conducted using ClinicalTrials.gov. DMD-related records were identified using the free-text terms “Duchenne muscular dystrophy,” “Duchenne,” and “DMD”; the search was not restricted by trial start year. Eligible records were interventional trials with a DMD-specific objective. Trial-level eligibility characteristics, study design, intervention type, trial phase, funding source, results posting, and start year were summarized. Era-wise comparisons were performed using the Fisher-Freeman-Halton exact test.

Results

A total of 232 interventional DMD trial records were included. Male-only eligibility was recorded for 198/232 trials (85.3%), and treatment was the primary purpose in 179/232 (77.2%). Drug interventions were the most frequent coded intervention category (101/232; 43.5%), Phase 2 was the largest phase category (58/232; 25.0%), and industry was the most common funder class (118/232; 50.9%). Results were posted on ClinicalTrials.gov for 76/232 trials (32.8%). The proportion of drug-intervention trials differed across start-year eras (p = 0.002). Biological interventions showed numerically greater representation in the most recent era, but the era-wise difference was not statistically significant (p = 0.420); industry sponsorship, Phase 3/4 status, randomization, and masking also did not differ significantly across eras.

Conclusions

ClinicalTrials.gov records show a predominantly treatment-oriented DMD research profile, with substantial industry involvement and frequent small-cohort studies. Therapeutic modalities are diverse, with biological and genetic approaches representing an important share of recent registered activity; however, most temporal comparisons of trial-design characteristics were not statistically significant. Limited registry results posting remains an important gap.

## Introduction

Duchenne muscular dystrophy (DMD) is one of the most severe inherited neuromuscular disorders of childhood, affecting approximately one in 3,500 to 5,000 live male births worldwide [1]. It is caused by pathogenic variants in the DMD gene that result in dystrophin deficiency or absence. Dystrophin is a structural protein that links the intracellular cytoskeleton of muscle fibers to the extracellular matrix. Progressive muscle degeneration leads to progressive weakness, loss of ambulation, respiratory insufficiency, and premature mortality [2]. Advances in understanding the pathophysiology of DMD have led to the development of diverse therapeutic approaches, including corticosteroids, exon-skipping therapies, nonsense mutation read-through agents, gene replacement strategies, and emerging cell-based therapies [3]. Consequently, clinical research on DMD has expanded substantially, reflecting the need to improve survival and functional outcomes. However, challenges related to equitable access to clinical research and the representation of diverse patient populations remain [4].

Despite the increasing number of DMD clinical trials, the overall clinical trial landscape remains incompletely characterized. Trial characteristics, including study phase, allocation, masking, enrollment, and outcome assessment, vary considerably [3]. The expanding range of interventions, including pharmacological agents, exon-skipping therapies, gene therapies, and other molecular approaches, has further increased the heterogeneity of the research landscape [5,6]. Differences in funding sources may also influence research priorities and trial conduct. Moreover, the underrepresentation of certain patient groups may limit the generalizability of clinical evidence [7]. Although previous studies have primarily focused on therapeutic efficacy and clinical outcomes, comprehensive registry-level evaluations simultaneously describing eligibility characteristics, study design, intervention type, trial phase, funding, results posting, and temporal patterns remain limited [6].

Therefore, a systematic assessment of the DMD clinical trial landscape is warranted to characterize research priorities and identify gaps in trial design, intervention development, and participant representation. ClinicalTrials.gov (https://ClinicalTrials.gov) provides a publicly accessible registry of individual clinical studies and structured trial-level fields that enable large-scale evaluation of these characteristics [8]. Review-focused databases were outside the study scope because the present study aimed to characterize individual registered trials rather than synthesize published studies or review protocols. ClinicalTrials.gov does not capture every trial conducted worldwide, and findings from this registry should therefore not be interpreted as a complete census of global DMD research activity. Accordingly, this study characterized interventional DMD trial records in ClinicalTrials.gov as of May 25, 2026, focusing on trial design, intervention type, phase, funding, results posting, and start-year patterns; the analysis concerns registered trial records rather than patient-level outcomes.

## Materials and methods

Study design and data sources

A retrospective, registry-based descriptive observational study was conducted using data from the U.S. National Library of Medicine’s ClinicalTrials.gov (https://ClinicalTrials.gov) database [8]. In practical terms, the unit of analysis was the registered trial record rather than the individual participant; thus, the analysis focused on clinical trial characteristics rather than patient-level outcomes. The analysis was intended to describe registry-record characteristics and temporal patterns; it was not a rapid or systematic review of published therapeutic efficacy studies.

Search strategy and eligibility criteria

ClinicalTrials.gov was searched on May 25, 2026, using the exact free-text terms “Duchenne muscular dystrophy,” “Duchenne,” and “DMD.” The terms were entered into the registry web search interface, and the retrieved records were combined to identify unique records using the NCT identifier. The original search workflow used the general free-text interface and did not document a separate AFR; therefore, no retrospective field restriction was imputed. No restriction was applied to trial start year, geographic location, sponsor, recruitment status, phase, enrollment size, or results-posting status at retrieval. The interventional design and DMD relevance were assessed during the eligibility screening. The search and eligibility criteria were defined before the final descriptive analysis.

Records were eligible if they were classified as interventional studies and focused on DMD-specific therapeutic, diagnostic, supportive, rehabilitative, or care-delivery interventions, regardless of recruitment status, trial phase, allocation, masking, sponsor type, geographic location, enrollment size, or results-reporting status. Records were assessed using the ClinicalTrials.gov identifier, study title, condition, study type, intervention, brief summary, eligibility criteria, and available registry information. We did not retain studies that focused exclusively on Becker muscular dystrophy, records without a DMD-specific objective, broad neuromuscular or muscular dystrophy studies without explicit DMD relevance, non-interventional or expanded-access records, duplicate or misaligned records, or records lacking sufficient information to confirm eligibility.

**Figure 1:**
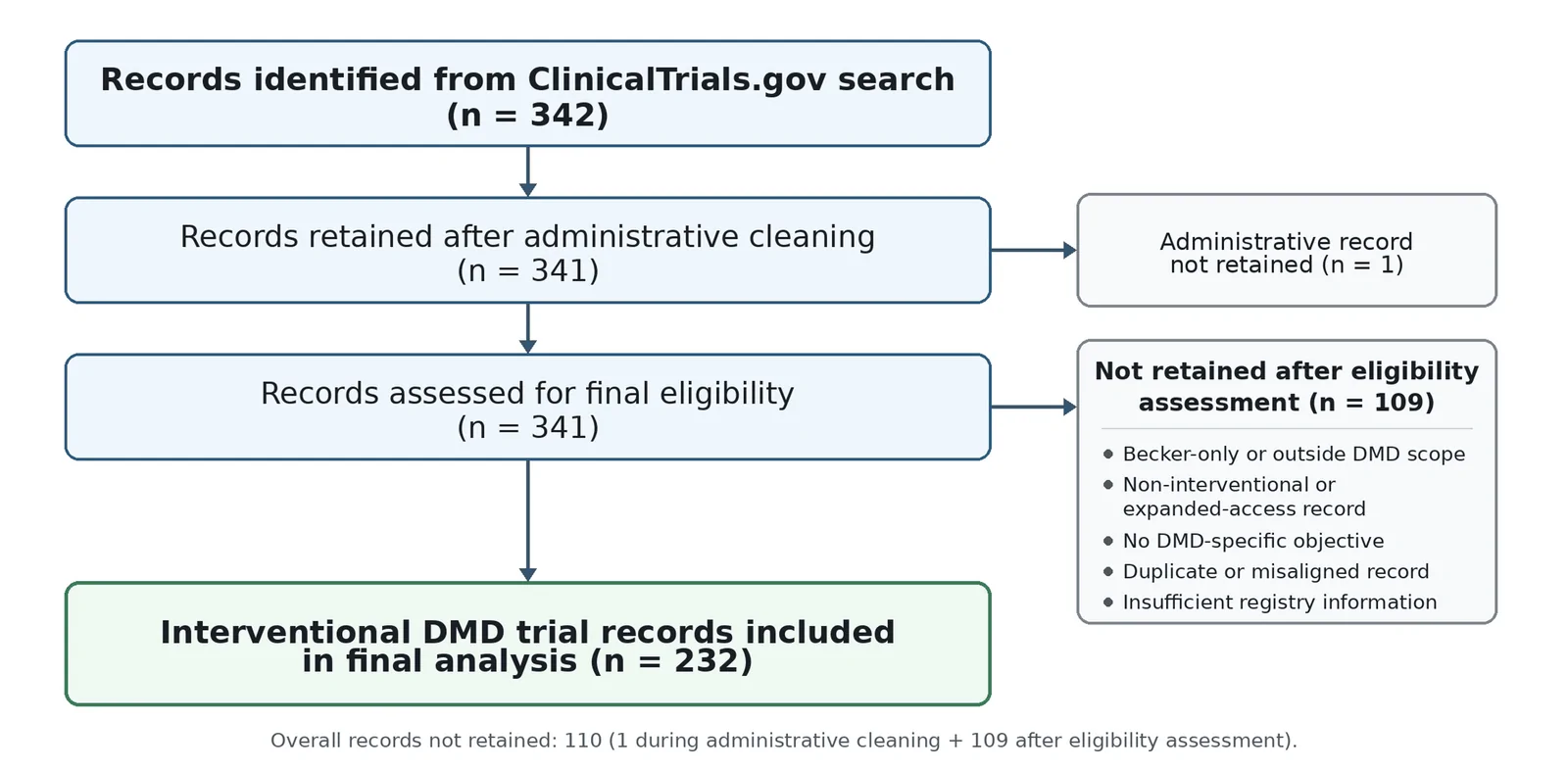
Study selection flow for interventional DMD trial records identified from ClinicalTrials.gov DMD: Duchenne muscular dystrophy

The initial search identified 342 records. One record was not retained during administrative cleaning, leaving 341 records for the final eligibility assessment. A further 109 records were not retained for the analytic dataset for one or more of the above-mentioned reasons, resulting in 232 eligible interventional DMD trial records for final analysis (Figure 1). The original screening worksheet did not preserve mutually exclusive numerical counts for each individual exclusion reason; therefore, rather than assigning retrospective reason-specific counts, Figure 1 reports the overall excluded count together with the documented exclusion categories.

Screening and data extraction

Screening and extraction were conducted using the study team’s predefined abstraction workflow rather than through formal duplicate independent screening of every record. Ambiguous eligibility or coding entries were rechecked against the corresponding ClinicalTrials.gov record before final classification. Trial-level data were extracted from the registry fields required for the prespecified descriptive objectives into an author-created abstraction template developed before the final analysis. This was an internal study workflow rather than a separately registered review protocol. Formal independent data extraction was not part of the original workflow.

The variables included trial start year, recruitment status, eligibility sex and age group, enrollment, allocation, masking, primary purpose, intervention type, trial phase, funder class, and results availability in the registry. Sex and age represented registry-defined eligibility criteria rather than the actual characteristics of enrolled participants. Enrollment reflected planned or actual enrollment as reported in the registry record.

Variable coding, denominators, and temporal analysis

Enrollment was categorized as ≤ 50, 51-100, 101-250, 251-500, or ≥ 500 participants. Allocation was categorized as randomized, non-randomized, or not applicable/not available. Masking was classified as none, single, double, triple, quadruple, or not available/unclear. Intervention types were categorized as drug, biological, genetic, device, procedure, radiation, dietary supplement, diagnostic test, behavioral, other, or not available/unclear. Funding sources were categorized as industry, National Institutes of Health (NIH), other government, or other; network-funded records were included in the “other” category.

The full final analytic cohort (N = 232) was used as the denominator for descriptive summaries, and missing, unavailable, or noninterpretable fields were retained as explicit not available/unclear categories where required. This approach makes the denominator visible and avoids implying that the full cohort is represented by available-case percentages.

Temporal comparisons were based on the registry-reported trial start year, not the registration or first-posted date. Trial start years were grouped into three prespecified pragmatic eras: ≤ 2010, 2011-2020, and ≥ 2021. These broad categories were chosen to contrast earlier registry activity, the 2011-2020 decade, and the most recent period while retaining adequate cell sizes for descriptive comparison. One trial with a missing start year was excluded from the era-wise analyses, leaving 231 records (≤ 2010, N = 30; 2011-2020, N = 124; ≥ 2021, N = 77). Because several 2 × 3 contingency tables contained small cell counts, Fisher-Freeman-Halton exact tests were used instead of relying on chi-square approximation assumptions. All tests were two-sided, and p < 0.05 was considered statistically significant.

Ethical considerations

This study used publicly available, de-identified trial-level registry data and did not involve individual participant-level data, protected health information, direct patient contact, or identifiable biospecimens; therefore, institutional ethics committee approval and informed consent were not required [9].

## Results

Study selection and eligibility criteria

A total of 342 clinical trial records related to DMD were initially identified. After administrative cleaning and the application of the eligibility criteria, 110 records were excluded, leaving 232 interventional clinical trial records for final analysis (Figure 1). The included trials represented 67.83% of the initially identified records.

Table 1 shows the participant eligibility characteristics. Male-only eligibility was recorded in 198 trials (85.3%), female-only eligibility in two (0.9%), and all-sex eligibility in 32 (13.8%). Child-only eligibility was the most common age category (n = 115; 49.6%), followed by combined child, adult, and older-adult eligibility (n = 66; 28.4%). Most trials planned enrollment of ≤ 50 participants (n = 171; 73.7%); enrollment was not available for two records (0.9%). These values describe the registry eligibility criteria and trial-level enrollment fields, not the actual demographic composition of the enrolled participants.

**Table 1:** Characteristics of included DMD clinical trial records (N = 232) Percentages are based on the full analytic cohort (N = 232). Eligibility sex and age are registry-defined eligibility criteria, not actual participant demographics. “Not available/unclear” indicates missing or noninterpretable registry fields DMD: Duchenne muscular dystrophy

Variable	N (%)
Participant eligibility	
Sex eligibility	
Male	198 (85.3)
Female	2 (0.9)
All	32 (13.8)
Age eligibility	
Adult	4 (1.7)
Adult and older adults	7 (3.0)
Child	115 (49.6)
Child and adult	40 (17.2)
Child, adult, and older adult	66 (28.4)
Planned enrollment	
≤ 50	171 (73.7)
51-100	27 (11.6)
101-250	27 (11.6)
251-500	5 (2.2)
≥ 500	0 (0.0)
Not available	2 (0.9)
Clinical trial characteristics	
Study status	
Completed	110 (47.4)
Recruiting/enrolling by invitation	29 (12.5)
Active, not recruiting	13 (5.6)
Not yet recruiting	8 (3.4)
Terminated	40 (17.2)
Withdrawn	7 (3.0)
Unknown	25 (10.8)
Allocation	
Randomized	80 (34.5)
Non-randomized	37 (15.9)
Not applicable/not available	115 (49.6)
Masking	
None	132 (56.9)
Single	10 (4.3)
Double	11 (4.7)
Triple	6 (2.6)
Quadruple	47 (20.3)
Not available/unclear	26 (11.2)
Primary purpose	
Treatment	179 (77.2)
Prevention	7 (3.0)
Diagnostic	6 (2.6)
Screening	2 (0.9)
Basic science	9 (3.9)
Health services research	3 (1.3)
Supportive care	11 (4.7)
Other	9 (3.9)
Not available/unclear	6 (2.6)
Results posted in ClinicalTrials.gov	
Yes	76 (32.8)
No	156 (67.2)

Trial design, purpose, and results posting

The most common status category was completed trials (n = 110; 47.4%), followed by terminated trials (n = 40; 17.2%). Randomized designs accounted for 80 trials (34.5%) and non-randomized designs for 37 (15.9%), whereas allocation was not applicable or unavailable for 115 records (49.6%). The most frequent masking category was unmasked trials (n = 132; 56.9%). Treatment was the primary purpose (n = 179; 77.2%). Results were posted in ClinicalTrials.gov for 76 trials (32.8%), whereas no registry-posted results were found in 156 (67.2%) trials.

Intervention type, phase, and funding

**Figure 2:**
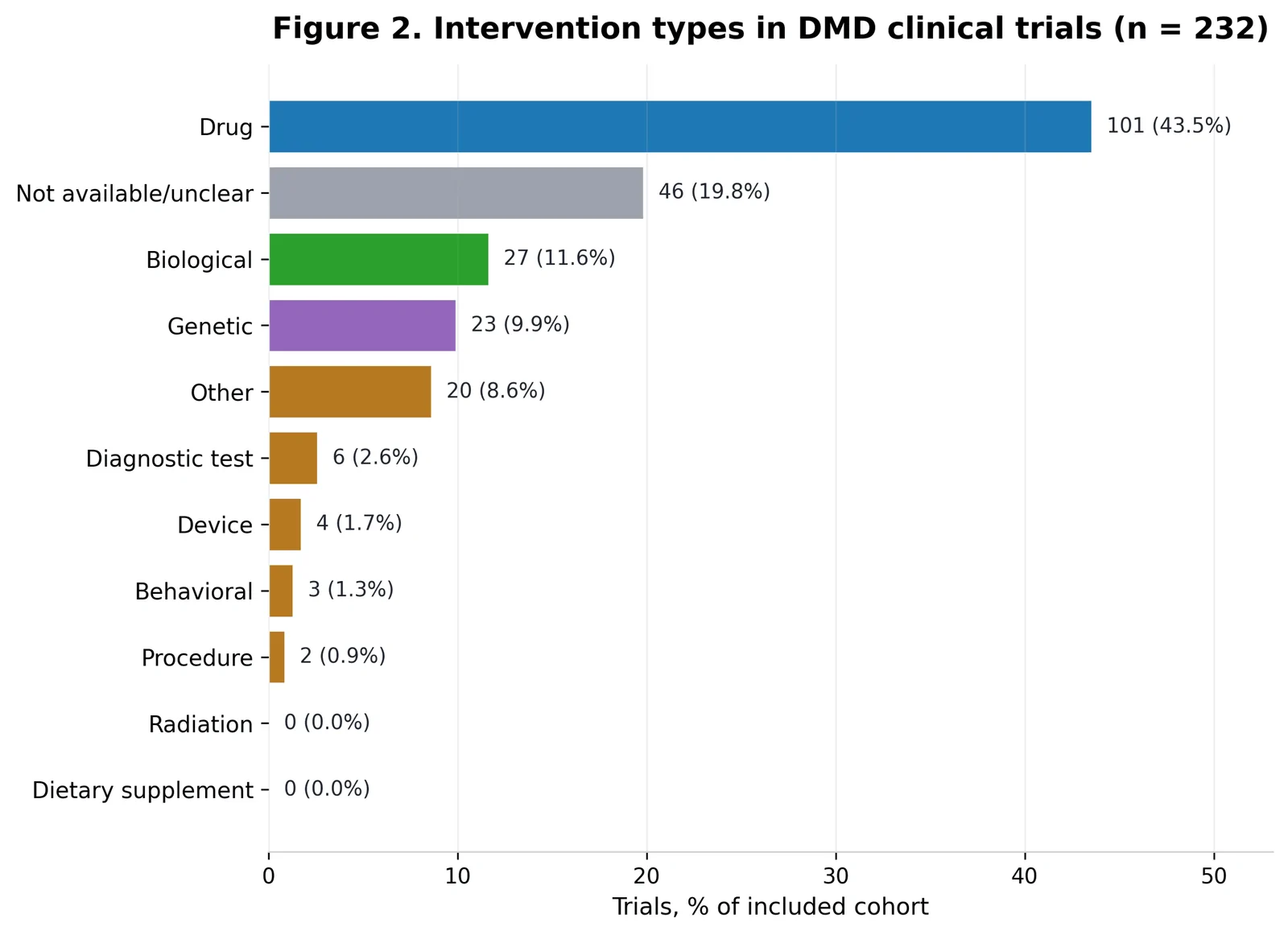
Intervention-type distribution among included DMD clinical trial records (N = 232) DMD: Duchenne muscular dystrophy

Table 2 presents the intervention types, trial phase, and funding. Drug interventions were the most frequent coded intervention category (n = 101; 43.5%), followed by biological interventions (n = 27; 11.6%) and genetic interventions (n = 23; 9.9%) (Figure 2). Intervention type was not available or remained unclear in 46 records (19.8%). Phase 2 trials were the largest phase category (n = 58; 25.0%), followed by Phase 3 and Phase 1/2 trials (n = 36; 15.5% each); phase was not applicable or not available in 55 records (23.7%). Industry was the most common funder class (n = 118; 50.9%), followed by other funding sources (n = 104; 44.8%).

**Table 2:** Distribution of intervention type, trial phase, and funding among included trial records (N = 232) Percentages are based on the full analytic cohort (N = 232). Network-funded records were grouped under “Other.” Genetic interventions were retained as a separate DMD-relevant category NIH: National Institutes of Health; DMD: Duchenne muscular dystrophy

Variable	N (%)
Intervention type	
Drug	101 (43.5)
Biological	27 (11.6)
Genetic	23 (9.9)
Device	4 (1.7)
Procedure	2 (0.9)
Radiation	0 (0.0)
Dietary supplement	0 (0.0)
Diagnostic test	6 (2.6)
Behavioral	3 (1.3)
Other	20 (8.6)
Not available/unclear	46 (19.8)
Clinical trial phase	
Early Phase 1	3 (1.3)
Phase 1	31 (13.4)
Phase 2	58 (25.0)
Phase 3	36 (15.5)
Phase 4	6 (2.6)
Phase 2/3	7 (3.0)
Phase 1/2	36 (15.5)
Not applicable/not available	55 (23.7)
Funder type	
Industry	118 (50.9)
NIH	2 (0.9)
Other government	8 (3.4)
Other	104 (44.8)

Era-wise comparisons

**Figure 3:**
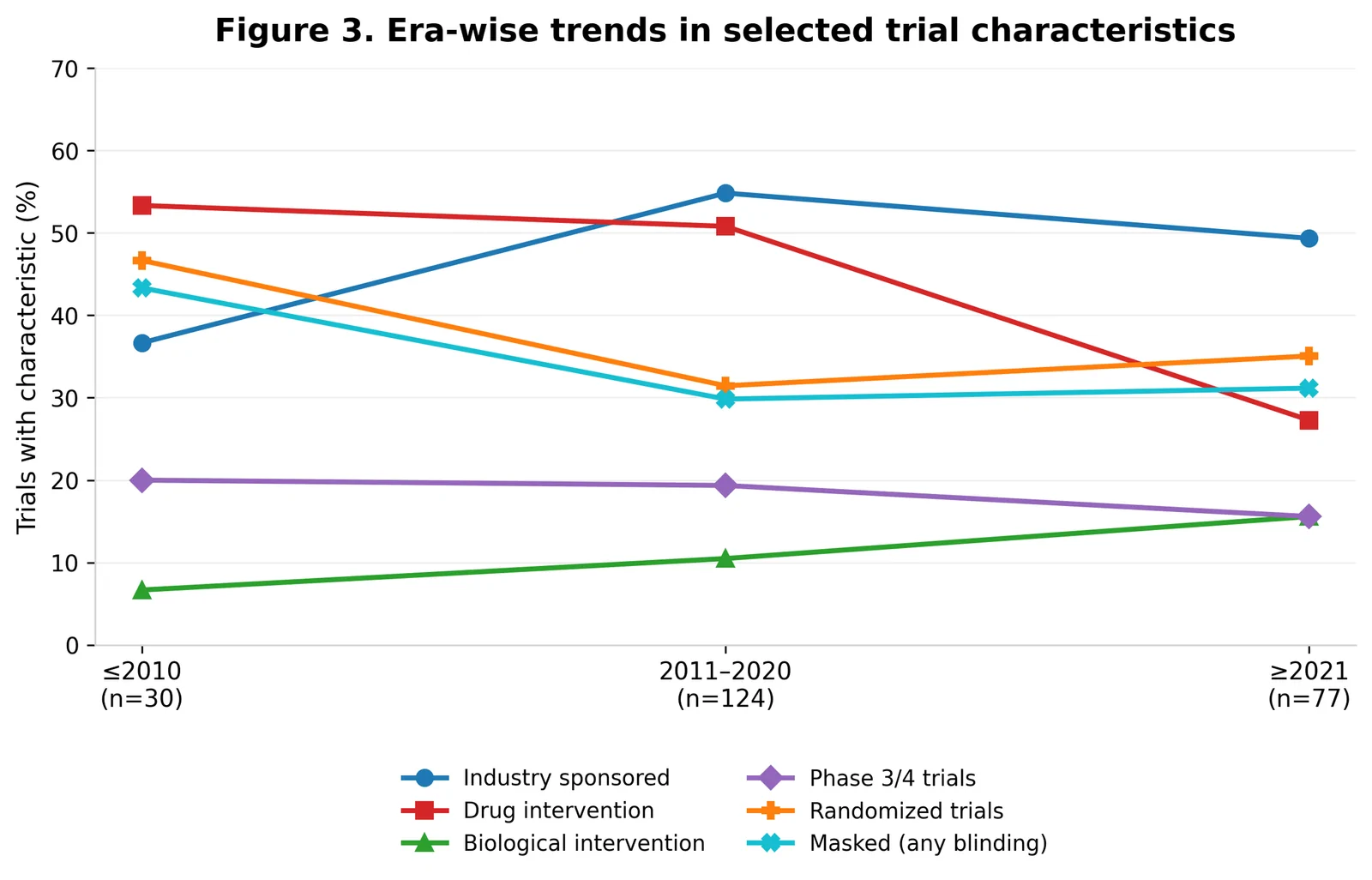
Era-wise proportions of selected trial characteristics One record with a missing start year was excluded from temporal analyses

Table 3 shows era-wise comparisons based on trial start year. The prevalence of drug intervention differed significantly across eras, decreasing from 16/30 trials (53.3%) in the ≤ 2010 era and 63/124 (50.8%) in 2011-2020 to 21/77 (27.3%) in the ≥ 2021 era (p = 0.002). Biological interventions showed numerically greater representation across the three eras (2/30 (6.7%), 13/124 (10.5%), and 12/77 (15.6%), respectively) (Figure 3), but this difference was not statistically significant (p = 0.420). Differences across eras were also not statistically significant for industry sponsorship (p = 0.195), phase 3/4 trials (p = 0.765), randomized design (p = 0.287), or any masking (p = 0.365).

**Table 3:** Era-wise comparison of selected characteristics of DMD clinical trial records DMD: Duchenne muscular dystrophy

Variable	≤ 2010 (N = 30), n/N (%)	2011-2020 (N = 124), n/N (%)	≥ 2021 (N = 77), n/N (%)	P-value
Industry-sponsored	11/30 (36.7)	68/124 (54.8)	38/77 (49.4)	0.195
Drug intervention	16/30 (53.3)	63/124 (50.8)	21/77 (27.3)	0.002
Biological intervention	2/30 (6.7)	13/124 (10.5)	12/77 (15.6)	0.42
Phase 3/4 trials	6/30 (20.0)	24/124 (19.4)	12/77 (15.6)	0.765
Randomized trials	14/30 (46.7)	39/124 (31.5)	27/77 (35.1)	0.287
Masked (any blinding)	13/30 (43.3)	37/124 (29.8)	24/77 (31.2)	0.365

## Discussion

This study characterized the interventional clinical trial landscape of DMD using ClinicalTrials.gov registry data. The trial landscape was dominated by treatment-focused studies, and drug interventions were the most frequent coded intervention category, with Phase 2 representing the largest phase category. Most trials had a planned enrollment of 50 participants or fewer. Randomized designs accounted for approximately one-third of all included records, while allocation was not applicable or unavailable in nearly half; therefore, the overall trial population should not be described as predominantly randomized. Unmasked trials were common. Industry was the leading funding class, and results were posted in ClinicalTrials.gov for only a minority of registered trials. Era-wise comparisons demonstrated a statistically significant difference in the proportion of drug-intervention trials, while the numerical increase in biological-intervention representation and the comparisons for industry sponsorship, Phase 3/4 status, randomization, and masking were not statistically significant.

Evaluating the DMD clinical trial landscape is important because the disease remains progressive and life-limiting despite advances in supportive care and therapeutic development [2,10]. Patients may experience progressive skeletal muscle weakness, loss of ambulation, respiratory complications, cardiomyopathy, and premature mortality. Characterizing how registered trials are distributed across intervention types, study phases, design characteristics, and funding sources can help identify gaps in the evidence-generation landscape represented in ClinicalTrials.gov. These registry-based observations should not, however, be interpreted as a complete assessment of all worldwide DMD research activity.

Drug-based interventions were the most frequent coded intervention category in this study (Table 2), consistent with the established role of pharmacological approaches in DMD management [11,12]. This predominance may reflect the earlier development and broader clinical feasibility of pharmacological therapies compared with newer biologic and genetic approaches. Biological and genetic interventions together also represented an important portion of coded trial activity, and the proportion of biological interventions was numerically higher in the ≥ 2021 era (Table 3). The era-wise difference for biological interventions was not statistically significant (p = 0.420), so this finding is best interpreted as an observed pattern rather than a confirmed temporal increase. In contrast, the proportion of drug-intervention records differed significantly by era (p = 0.002). This finding demonstrates a change in the registry composition with respect to drug-intervention trials but does not establish that the overall therapeutic landscape underwent a statistically confirmed transformation. The broader emergence of disease-mechanism-directed approaches, including exon-skipping and other gene-targeted strategies, is consistent with the existing therapeutic literature [13].

Phase 2 trials were the most frequently represented phase (Table 2), indicating that midstage classifications are common among the registered trial records in this dataset. Previous analyses of DMD therapeutics have similarly described substantial early- and intermediate-stage clinical development activity [14,15]. However, registry phase distributions should not be equated with the current development pipeline of unique therapeutic candidates: multiple trials may evaluate the same intervention, and the registry phase assigned to an individual record does not necessarily represent the current development status of a therapeutic platform. The era-wise proportion of Phase 3/4 records did not differ significantly (p = 0.765).

Randomization was present in 80 of 232 records (34.5%) and was an important design characteristic. Randomization can reduce selection bias and strengthen causal inference, but it does not by itself establish overall methodological quality. Allocation concealment, sample size, masking, outcome selection, statistical power, and completeness of follow-up also contribute to trial rigor. Unmasked studies remained common, and masking may be practically difficult for invasive procedures, gene-based therapies, distinct administration routes, or interventions with recognizable effects [16]. Randomization and masking did not show significant era-wise differences in the present analysis (p = 0.287 and p = 0.365, respectively).

Treatment was the predominant primary purpose of registered trials (Table 1), consistent with the urgent need for disease-modifying therapies in DMD [17]. However, the limited number of studies focused on prevention, screening, diagnosis, supportive care, and health services research suggests that broader care needs may receive comparatively less attention within this registry-defined interventional landscape. Given the multisystem nature of DMD, future research should also address respiratory and cardiac outcomes, functional preservation, quality of life, rehabilitation, and patient-centered outcomes.

Industry was the most common funding source (Table 2), consistent with previous reports describing substantial industry involvement in DMD therapeutic development [18]. The present study demonstrates the frequency of industry sponsorship in the registry dataset; it did not evaluate whether industry sponsorship accelerates therapeutic development, improves trial completion, or affects clinical success. Interpretations about the role of industry beyond sponsorship frequency therefore derive from the existing literature rather than from the present data. Collaboration among industry, academic institutions, public agencies, and nonprofit organizations remains relevant to supporting a balanced research agenda [19]. Industry sponsorship itself did not differ significantly across eras (p = 0.195).

Results were posted in ClinicalTrials.gov for only 76 of 232 included trial records (32.8%). This finding specifically refers to results reporting in the registry and should not be interpreted as the proportion of trials with a scientific publication available elsewhere. Limited registry results posting may reduce transparency and hinder evidence synthesis, particularly in rare diseases where eligible participant populations are limited. Improving the completeness and timeliness of registry results reporting would strengthen accountability and facilitate interpretation of the cumulative evidence base.

Overall, the findings indicate that DMD clinical research represented in ClinicalTrials.gov remains predominantly treatment-oriented, with drug interventions, Phase 2 studies, and substantial industry sponsorship forming important components of the registry landscape, while biological and genetic approaches are also represented. Future research should prioritize diverse therapeutic strategies, broader clinical outcomes, improved reporting transparency, and trial designs that address the multisystem and long-term needs of individuals with DMD.

Limitations

This study was limited to trials registered in ClinicalTrials.gov and may not capture studies registered exclusively in other national or international trial registries; accordingly, it does not represent a complete global census of DMD research. The original search workflow documented the free-text terms and retrieval date but not a separate advanced-field selector, which limits exact interface-level replication of the historical search. Registry-based analyses depend on the completeness, accuracy, and updating of sponsor- or investigator-submitted information, and changes in registration practices over time may influence temporal comparisons. The broad start-year eras were selected for descriptive interpretability and cell size rather than to represent distinct time epochs.

The analysis included registered trial records rather than unique therapeutic candidates, so multiple records may represent the same intervention. The study could not assess the efficacy, safety, or patient-level outcomes of individual interventions. Additionally, the original workflow did not use formal duplicate independent screening or duplicate independent data extraction for every record, and the screening worksheet did not preserve mutually exclusive counts for each individual exclusion reason. These features may allow classification error and limit the granularity of the selection audit. These limitations should be considered when interpreting descriptive patterns and temporal comparisons.

## Conclusions

This study provides an overview of the interventional clinical trial landscape in DMD represented in ClinicalTrials.gov. The registry dataset demonstrates a predominantly treatment-focused research agenda, frequent small-cohort studies, a large Phase 2 component, and substantial industry sponsorship. Randomized designs were present in a meaningful proportion of records, but they did not constitute the majority of all included trials, and unmasked studies were common. Biological and genetic approaches represented an important share of recent registered activity; however, the observed numerical increase in biological interventions was not statistically significant, and most temporal comparisons of trial-design characteristics were also not statistically significant. The significant era-wise difference in drug-intervention prevalence should therefore be interpreted specifically rather than as evidence of broad methodological improvement. Gaps remain in registry results posting and in the representation of diagnostic, preventive, supportive-care, and broader patient-centered interventions. Future studies should integrate patient-level characteristics, treatment outcomes, diverse populations, and long-term follow-up to strengthen the clinical relevance and generalizability of evidence in DMD.

## Appendices

This appendix documents the search and screening procedure reflected in the revised manuscript. It is provided to improve reproducibility without retrospectively inventing methodological details that were not recorded in the original workflow.

Registry and date

ClinicalTrials.gov; search performed 25 May 2026.

Free-text terms

“Duchenne muscular dystrophy,” “Duchenne,” and “DMD.”

Search interface

General ClinicalTrials.gov web search interface. The original workflow did not document a separate advanced-field selector (for example, a restriction only to Condition, Title, or Other Terms), so no such restriction is retrospectively claimed.

Retrieval filters

No trial-start-year, geographic, sponsor, recruitment-status, phase, enrollment-size, or results-posting restriction was applied at retrieval. Interventional design and DMD relevance were determined during eligibility screening.

Deduplication

Retrieved records were combined, and unique records were identified using the NCT identifier.

Eligibility review fields

NCT identifier, study title, condition, study type, intervention, brief summary, eligibility criteria, and other available registry information.

Included records

Interventional studies with a DMD-specific therapeutic, diagnostic, supportive, rehabilitative, or care delivery objective, irrespective of recruitment status, phase, allocation, masking, sponsor type, geography, enrollment size, or registry results-posting status.

Not retained

Becker-only studies, records without a DMD-specific objective, broad neuromuscular or muscular dystrophy studies without explicit DMD relevance, non-interventional or expanded-access records, duplicate or misaligned records, and records with insufficient information to confirm eligibility.

Selection accounting

342 records were initially identified. One record was removed during administrative cleaning, 341 underwent final eligibility assessment, and 109 additional records were not retained, leaving 232 records for analysis. Because the original worksheet did not preserve mutually exclusive counts for every individual exclusion reason, reason-specific numerical counts are not retrospectively assigned.

Screening and extraction workflow

The study team used an author-created abstraction workflow defined before the final descriptive analysis. The original workflow did not include formal duplicate independent screening and extraction of every record; ambiguous entries were rechecked against the source ClinicalTrials.gov record.

Temporal variable

Era-wise analyses used registry-reported trial start year, not registration date or first-posted date. One record lacked start year and was excluded only from temporal analyses.

Era definitions

≤ 2010 (N = 30), 2011-2020 (N = 124), and ≥2021 (N = 77). These pragmatic categories contrast earlier registry activity, the 2011-2020 decade, and the most recent period while maintaining usable cell sizes.

Statistical testing

Because several 2 × 3 contingency tables had small cell counts, two-sided Fisher-Freeman-Halton exact tests were used for era-wise comparisons; p < 0.05 was considered statistically significant.

## References

[1] Emery AE (2002). The muscular dystrophies. Lancet.

[2] Birnkrant DJ, Bushby K, Bann CM (2018). Diagnosis and management of Duchenne muscular dystrophy, part 1: diagnosis, and neuromuscular, rehabilitation, endocrine, and gastrointestinal and nutritional management. Lancet Neurol.

[3] Duan D, Goemans N, Takeda S, Mercuri E, Aartsma-Rus A (2021). Duchenne muscular dystrophy. Nat Rev Dis Primers.

[4] Birnkrant DJ, Bushby K, Bann CM (2018). Diagnosis and management of Duchenne muscular dystrophy, part 2: respiratory, cardiac, bone health, and orthopaedic management. Lancet Neurol.

[5] Takeda S, Clemens PR, Hoffman EP (2021). Exon-skipping in Duchenne muscular dystrophy. J Neuromuscul Dis.

[6] Sheikh O, Yokota T (2021). Value: Restoring protein expression in neuromuscular conditions: a review assessing the current state of exon skipping/inclusion and gene therapies for Duchenne muscular dystrophy and spinal muscular atrophy. BioDrugs.

[7] Birnkrant DJ, Bushby K, Bann CM (2018). Diagnosis and management of Duchenne muscular dystrophy, part 3: primary care, emergency management, psychosocial care, and transitions of care across the lifespan. Lancet Neurol.

[8] (2026). ClinicalTrials.gov. U.S. National Library of Medicine. https://clinicaltrials.gov/.

[9] (2026). U.S. Department of Health and Human Services, Office for Human Research Protections: 45 CFR 46. https://www.hhs.gov/ohrp/regulations-and-policy/regulations/45-cfr-46/index.html.

[10] Heydemann A, Siemionow M (2023). A brief review of Duchenne muscular dystrophy treatment options, with an emphasis on two novel strategies. Biomedicines.

[11] Komaki H (2025). Duchenne muscular dystrophy: evolving therapeutic strategies and multidimensional evaluation approaches. Brain Dev.

[12] Bez Batti Angulski A, Hosny N, Cohen H, Martin AA, Hahn D, Bauer J, Metzger JM (2023). Duchenne muscular dystrophy: disease mechanism and therapeutic strategies. Front Physiol.

[13] Wilton-Clark H, Yokota T (2023). Biological and genetic therapies for the treatment of Duchenne muscular dystrophy. Expert Opin Biol Ther.

[14] Verhaart IE, Aartsma-Rus A (2019). Therapeutic developments for Duchenne muscular dystrophy. Nat Rev Neurol.

[15] Tang A, Yokota T (2024). Duchenne muscular dystrophy: promising early-stage clinical trials to watch. Expert Opin Investig Drugs.

[16] Merlini L, Sabatelli P (2015). Improving clinical trial design for Duchenne muscular dystrophy. BMC Neurol.

[17] Shieh PB (2015). Duchenne muscular dystrophy: clinical trials and emerging tribulations. Curr Opin Neurol.

[18] Creasey M, Thangarajh M (2025). Inequitable racial and ethnic representation in Duchenne muscular dystrophy clinical trials. Neuropediatrics.

[19] Bendixen RM, Morgenroth LP, Clinard KL (2016). Engaging participants in rare disease research: a qualitative study of Duchenne muscular dystrophy. Clin Ther.

